# Functional connectivity of the amygdala subregions and the antidepressant effects of repeated ketamine infusions in major depressive disorder

**DOI:** 10.1192/j.eurpsy.2024.1744

**Published:** 2024-04-04

**Authors:** Haiyan Liu, Chengyu Wang, Xiaofeng Lan, Weicheng Li, Fan Zhang, Zhibo Hu, Yanxiang Ye, Yuping Ning, Yanling Zhou

**Affiliations:** 1 Department of Child and Adolescent Psychiatry, Affiliated Brain Hospital of Guangzhou Medical University, Guangzhou, China; 2 Key Laboratory of Neurogenetics and Channelopathies of Guangdong Province and the Ministry of Education of China, The Second Affiliated Hospital, Guangzhou Medical University, Guangzhou, China; 3 Guangdong Engineering Technology Research Center for Translational Medicine of Mental Disorders, Guangzhou, China; 4 Department of Psychology, The First School of Clinical Medicine, Southern Medical University, Guangzhou, China

**Keywords:** amygdala subregion, antidepressant, functional connectivity, ketamine, major depressive disorder

## Abstract

**Background:**

Amygdala subregion-based network dysfunction has been determined to be centrally implicated in major depressive disorder (MDD). Little is known about whether ketamine modulates amygdala subarea-related networks. We aimed to investigate the relationships between changes in the resting-state functional connectivity (RSFC) of amygdala subregions and ketamine treatment and to identify important neuroimaging predictors of treatment outcomes.

**Methods:**

Thirty-nine MDD patients received six doses of ketamine (0.5 mg/kg). Depressive symptoms were assessed, and magnetic resonance imaging (MRI) scans were performed before and after treatment. Forty-five healthy controls underwent one MRI scan. Seed-to-voxel RSFC analyses were performed on the amygdala subregions, including the centromedial amygdala (CMA), laterobasal amygdala (LBA), and superficial amygdala subregions.

**Results:**

Abnormal RSFC between the left LBA and the left precuneus in MDD patients is related to the therapeutic efficacy of ketamine. There were significant differences in changes in bilateral CMA RSFC with the left orbital part superior frontal gyrus and in changes in the left LBA with the right middle frontal gyrus between responders and nonresponders following ketamine treatment. Moreover, there was a difference in the RSFC of left LBA and the right superior temporal gyrus/middle temporal gyrus (STG/MTG) between responders and nonresponders at baseline, which could predict the antidepressant effect of ketamine on Day 13.

**Conclusions:**

The mechanism by which ketamine improves depressive symptoms may be related to its regulation of RSFC in the amygdala subregion. The RSFC between the left LBA and right STG/MTG may predict the response to the antidepressant effect of ketamine.

## Introduction

Major depressive disorder (MDD) is a common mental disorder characterized by a high incidence, high recurrence rate, and high disability rate [1, 2]. However, the effect of evidence-based treatment is not ideal at present [3]. Ketamine, an *N*-methyl-d-aspartic acid receptor antagonist, has attracted much attention because of its rapid antidepressant effect. Ketamine has been shown to be effective in treating adult patients with resistant depression. Several studies have suggested that repeated intravenous infusions of ketamine over 2–4 weeks may be more effective and may last longer than a single dose [4, 5]. However, due to the heterogeneity of ketamine treatment, nearly 40% of MDD patients do not respond to repeated infusions of ketamine, underscoring the need to find the best neural predictor of symptom improvement.

With the development of brain imaging technology, some studies have explored the biological indicators that can predict the treatment response of MDD patients from the perspective of resting-state functional connectivity (RSFC). The main feature of MDD is emotional processing dysfunction. Many studies have shown that the functional network of the amygdala, which is the center of emotional processing, is abnormal [6, 7]. Different antidepressant treatments have been found to be related to the regulation of the functional network of the amygdala [8, 9]. Our previous study confirmed that ketamine can change the functional coupling of the amygdala: the RSFC between the left amygdala and the left medial superior frontal gyrus of MDD patients increased significantly after six ketamine infusions, and the baseline RSFC between the amygdala and right putamen could predict the antidepressant effects of ketamine [10]. However, these studies regard the amygdala as a homogeneous whole, ignoring its heterogeneity.

The amygdala is a nuclear complex with different structures and functions. According to the characteristics of cell structure, the amygdala can be divided into three functional subregions: the laterobasal amygdala (LBA) receives information from the cortex and subcortex [11, 12], the centromedial amygdala (CMA) integrates information from other subregions of the amygdala and outputs the information into other brain regions, such as the brainstem and striatum [12–14], and the superficial amygdala (SFA) is responsible for social information processing [12, 15]. Some studies have shown that the RSFCs of the amygdala subregions in MDD patients are abnormal [16, 17]. Studies have reported that the intensity of RSFC between the CMA and rostral anterior cingulate and between the CMA and insula are associated with depressive symptoms in MDD patients [18, 19]. Luo et al. also found that RSFC between the left CMA and the left insula could modulate the relationship between childhood maltreatment and depression and trait anxiety levels [20]. These studies revealed a link between RSFC in the amygdala subregions and depressive symptoms.

A few studies have shown that antidepressant treatment can modulate the functional network of the amygdala subregion. One study reported that the strength of the RSFC between the left SFA and the left posterior fusiform gyrus increased with improvements in depressive symptoms after electroconvulsive therapy in MDD patients [21]. Additionally, another study showed that the baseline RSFC of the left LBA and left precuneus in treatment responders was stronger than that in nonresponders in patients with anxiety-related depression, and the RSFC between the left LBA and left precuneus could predict treatment outcome [22]. However, the role of RSFC in the amygdala subregion during ketamine treatment remains unclear.

Therefore, we conducted seed-to-voxel RSFC analysis between amygdala subregions and whole-brain voxels to investigate the relationships between changes in the RSFC of amygdala subregions and improvements in depressive symptoms after repeated ketamine infusions in MDD patients. We also compared changes in the RSFC of the amygdala subregions between responders and nonresponders over time (before and after ketamine treatment) to identify ketamine-induced changes in the RSFC specific to responders. In addition, we investigated whether the difference in the baseline RSFC of the amygdala subregion between responders and nonresponders was related to improvements in depressive symptoms and explored the potential of these differences to predict the antidepressant efficacy of ketamine.

## Methods

### Participants

Participants were recruited from a clinical trial that explored the antidepressant effects of repeated ketamine treatment on MDD patients. This study was carried out in accordance with the Helsinki Declaration of Ethical Principles and approved by the Clinical Research Ethics Committee of the Affiliated Brain Hospital of Guangzhou Medical University. All participants provided informed consent before entering the trial. The inclusion criteria for patients were as follows: (1) aged 18–65 years; (2) met the Diagnostic and Statistical Manual of Mental Diseases-5 (DSM-5, Structured Clinical Interview for DSM-5 [SCID]) [23] standard of MDD with no psychotic symptoms; (3) had a 17-item Hamilton Depression Scale (HAMD-17) score ≥ 17 [24]; and (4) exhibited suicidal ideation (confirmed by the Beck Scale for Suicidal Ideation (SSI) – Part I, with a score ≥ 2 at screening) [25] or experienced treatment resistance, defined as the failure of two or more adequate antidepressant trials. The exclusion criteria were as follows: (1) had any other psychiatric diagnosis according to the DSM-5 diagnostic criteria, such as bipolar disorder or schizophrenia; (2) had severe medical or neurological illness or severe head trauma; and (3) had substance dependence. Moreover, 45 healthy controls (HCs) who met the same exclusion criteria and, according to the SCID, had no previous mental disorders were recruited from the community.

### Procedures

All MDD patients received a 40-minute open-label infusion of ketamine (0.5 mg/kg, diluted in saline) three times a week for 2 weeks. The detailed research process, including safety monitoring, has been described in our previous studies [26, 27]. There were no restrictions on the use of psychiatric medications in MDD patients during the study period, but if patients were taking psychotropic medications at screening, a stable dose of ≥4 weeks had to be achieved before ketamine infusion, and patients continued to receive the same regimen and dose throughout the study period.

MDD patients were assessed for depressive symptoms 24 hours before the first ketamine infusion (baseline), 24 hours after the sixth ketamine infusion (Day 13), and then 1 week (Day 19) after the sixth ketamine infusion. All MDD patients underwent resting state-functional magnetic resonance imaging (rs-fMRI) scans at baseline and Day 13, whereas HCs underwent rs-fMRI scans only at baseline.

### Rating scales

The severity of depressive symptoms was assessed using the HAMD-17. The higher the score on this scale was, the more severe the depressive symptoms were. The reduction rate of the HAMD-17 score (ΔHAMD-17%) was used to assess the antidepressant effects of ketamine. The reduction rate of the HAMD-17 score was calculated as follows: pretreatment score minus posttreatment score, then divided by the pretreatment score, and finally multiplied by 100%. Responder status was defined as a ΔHAMD-17% ≥ 50% on Day 13.

### MRI acquisition

All rs-fMRI data were acquired by a 3.0-T Philips Achieva MRI scanner (Philips, the Netherlands) with eight-channel phased-array head coils and were acquired by using a gradient echo-planar imaging (EPI) sequence with the following parameters: echo time = 30 ms; repetition time = 2,000 ms; flip angle = 90°; 33 slices; matrix = 64 × 64; field-of-view = 220 × 220 × 150 mm^3^; voxel size = 3.44 × 3.44 × 4 mm^3^; gap = 0.6 mm; and number of signal averages = 1. The resting fMRI scan lasted 8 minutes, and 240 volumes were acquired. All subjects were required to keep their eyes closed and remain awake, avoiding systematic thinking during the scans.

### Image preprocessing

All fMRI data were preprocessed using the Data Processing and Analysis of Brain Imaging (DPABI, version 5.0) toolbox (http://rfmri.org/DPARSF) in MATLAB R2019b (MathWorks, Natick, MA). First, we discarded the first 10 time points and performed slice timing and head motion corrections (maximum head motion parameters of >2 mm translation and/or > 2.0° rotation were excluded). Then, EPI templates were used for spatial normalization, and a 4°mm full width at half maxima isotropic Gaussian kernel was used for smoothing. Linear and quadratic trends were removed, and linear regression was used to remove nuisance signals from 24-parameter head motion profiles, white matter signals, cerebrospinal fluid signals, and global signals. Finally, we filtered at 0.01–0.1 Hz and “scrubbed” one time point before and one time point after bad images, whose frame displacement > 0.5.

### Definition of region of interest and RSFC

Similar to previous studies, we used the statistical parametric mapping (SPM) Anatomy Toolbox (www.fz-juelich.de/inm/inm-1/DE/Forschung/_docs/SPMAnatomyToolbox/SPMAnatomyToolbox_node.html) based on probabilistic maps from the JuBrain Cytoarchitectonic Atlas to obtain bilateral amygdala subregions [28, 29]. The amygdala was divided into three subregions: the LBA, CMA, and SFA. Then, whole-brain voxel-level RSFC of each amygdala subregion was mapped for all subjects. The main steps were as follows: (1) calculating the average time series of each amygdala subregion; (2) calculating the Pearson correlation coefficient (*r*-value) between the average time series of each amygdala subregion and that of each voxel in the rest of the brain; and (3) converting the *r-*values to *z*-values by Fisher’s transformation to improve normality.

### Statistical analysis

Two independent samples *t-*tests or chi‐squared tests were applied to determine whether there were differences in the baseline demographic data between the MDD patients and HCs or between the responders and nonresponders.

First, two-sample independent *t-*tests in each amygdala subregion were performed to explore the differences in RSFC between MDD patients and HCs by using the SPM 12 toolbox (https://www.fil.ion.ucl.ac.uk/spm), controlling for age, sex, and head motion. According to normality, Pearson correlation or Spearman correlation analysis was conducted to identify the associations between the significant differences in RSFC identified above and the reduction rate of the HAMD-17 score.

Second, we used a flexible factorial design that included two factors, group (responders and nonresponders) and time (baseline and posttreatment), in SPM12 (https://www.fil.ion.ucl.ac.uk/spm/). The group × time interaction was calculated to explore response-related RSFC changes, with age, gender, head motion, and the medication load index as covariates. Additionally, two-sample independent *t*-tests in each amygdala subregion were performed to explore the differences in the RSFC between responders and nonresponders. Bivariate correlation analyses were then conducted to identify the associations between the significant differences in the RSFC reported above and the reduction rate of HAMD-17 score and between the changes in RSFC reported above after treatment and the reduction in depressive symptoms. Receiver operating characteristic (ROC) curve analysis was performed to determine whether the significant difference in the RSFC between responders and nonresponders could predict the response to ketamine.

All resulting group-level analyses had a threshold of *p* < 0.05 at the cluster level using false discovery rate (FDR) correction at a height threshold of *p* < 0.001.

All statistical analyses, other than the computation of three‐dimensional RSFC maps, were performed using the Statistical Package for Social Sciences (SPSS 25.0). *P* < 0.05 was considered to indicate statistical significance.

## Results

### Demographics of participants

**Figure 1.**
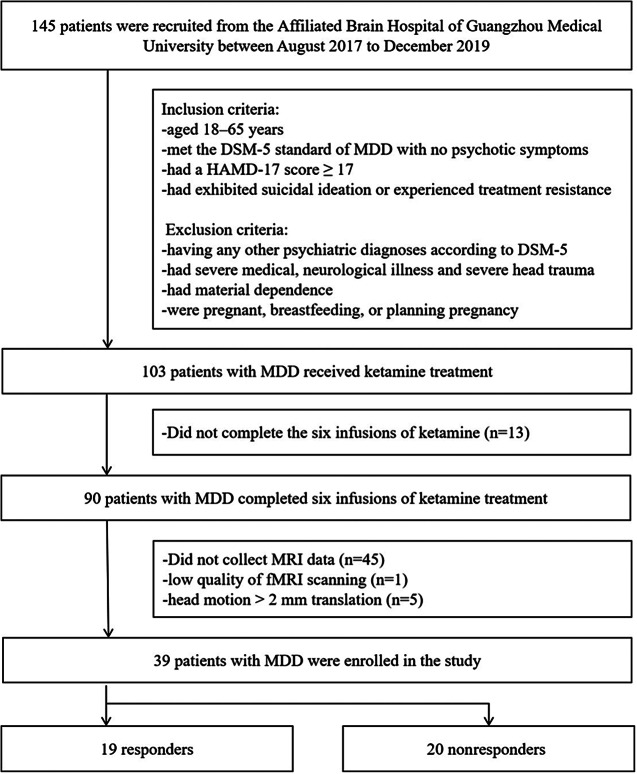
Flowchart. DSM-5, diagnostic and statistical manual of mental diseases-5; fMRI, functional magnetic resonance imaging; HAMD-17, 17-item Hamilton Depression Scale; MDD, major depressive disorder; MRI, magnetic resonance imaging.

A total of 39 MDD patients were included in this study. Among these, 19 patients (48.72%) were classified as responders, whereas 20 patients (51.28%) were defined as nonresponders. The demographic and clinical characteristics are described in Table 1. The flowchart is shown in Figure 1.

**Table 1.** Demographic and clinical characteristics

	MDD	Responders	Nonresponders	HCs	HCs versus MDD	Responder versus nonresponders
Characteristic	(*n* = 39)	(*n* = 19)	(*n* = 20)	(*n* = 45)	*p*-value	*p*-value
Age (years)	36.46 ± 12.11	40.58 ± 11.11	32.55 ± 11.96	31.40 ± 7.98	0.031*	0.037*
Gender (% females)^a^	24 (61.54%)	11 (57.89%)	13 (65.00%)	27 (60.00%)	0.886	0.648
Head motion	0.05 ± 0.02	0.06 ± 0.03	0.04 ± 0.01			0.039*
Education (years)	11.82 ± 3.28	11.63 ± 2.99	12.00 ± 3.60			0.731
BMI (kg/m^2^)	22.97 ± 3.19	23.68 ± 2.62	22.30 ± 3.58			0.180
Duration of illness (months)	84.08 ± 80.01	103.21 ± 84.50	65.90 ± 72.97			0.148
First episode (yes)^a^	15 (38.46%)	6 (31.58%)	9 (45.00%)			0.389
Age of onset (years)	29.03 ± 11.43	31.68 ± 11.01	26.50 ± 11.51			0.160
Psychiatric comorbidity^a^	6 (15.38%)	2 (10.50%)	4 (20.00%)			0.661
Baseline HAMD–17	23.23 ± 4.55	22.37 ± 2.67	24.05 ± 5.76			0.249
Use of drugs						
Medication load index	4.08 ± 1.75	4.05 ± 1.68	4.10 ± 1.86			0.934
SSRI	26	12	14			
SNRI	13	5	8			
NaSSA	6	3	3			
Tricyclic antidepressants	2	1	1			
Other	2	2	0			
Antidepressant monotherapy	25	11	14			
Two antidepressants	12	6	6			
Lithium	2	1	1			
Antipsychotics	25	11	14			
Benzodiazepines	21	11	10			

Abbreviations: BMI, body mass index; HAMD-17: 17-item Hamilton Depression Rating Scale; HCs, healthy controls; MDD, major depressive disorder; NaSSA, norepinephrine and specific serotonergic inhibitor; SNRI, selective serotonin-norepinephrine reuptake inhibitor; SSRI, selective serotonin reuptake inhibitor.

a *χ*2 test of continuity correction.

* *p* < 0.05.

### Differences in the RSFC of the amygdala subregion between HCs and MDD patients

**Figure 2.**
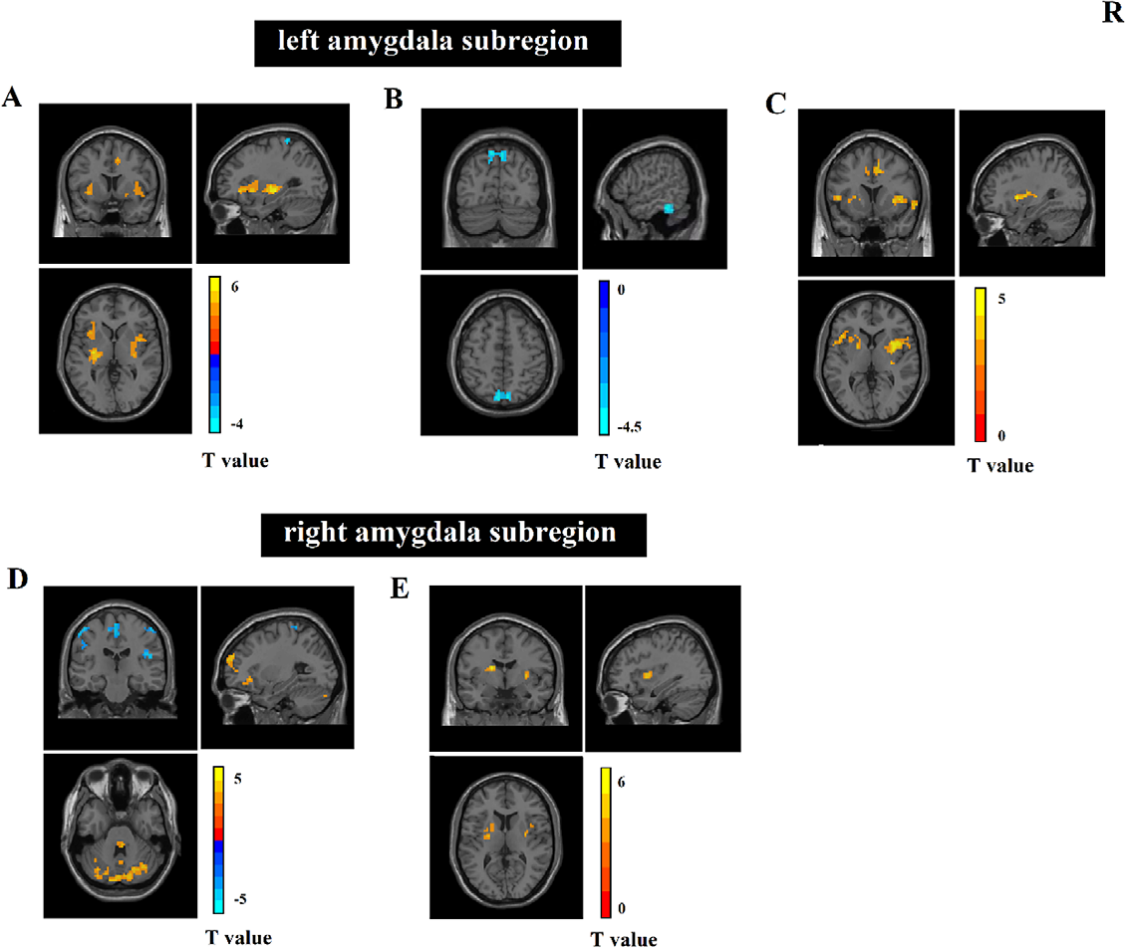
(A–E) Differences in the RSFC of amygdala subregions between HCs and MDD patients. HCs, healthy controls; MDD, major depressive disorder; RSFC, resting-state functional connectivity.

For each of the six a priori-defined amygdala subregion seeds, we assessed and compared whole-brain RSFCs between MDD patients and HCs. Compared to that in HCs, MDD patients displayed hyper-connectivity between the left CMA and the left postcentral gyrus. In addition, the MDD patients showed hypo-connectivity between the left CMA and the bilateral insula, left putamen, and right supplementary motor area (Figure 2 and Supplementary Table 1).

Compared to that in HCs, hyper-connectivity in MDD patients was shown between the left LBA and some clusters of the left precuneus and right inferior temporal gyrus (Figure 2B and Supplementary Table 1).

Abnormal RSFC is also presented in the left SFA. Compared to that in HCs, hypo-connectivity was shown between the left SFA and some clusters of the region in MDD patients, including the bilateral putamen and the left opercular part inferior frontal gyrus (IFGoperc), and right median cingulate and paracingulate gyri (DCG) (Figure 2C and Supplementary Table 1).

Compared to that in HCs, MDD patients displayed hyper-connectivity between the right CMA and some clusters of the bilateral postcentral gyrus, left paracentral lobule (PCL), right rolandic operculum, and right superior occipital gyrus. In addition, MDD patients exhibited hypo-connectivity between the left orbital part inferior frontal gyrus (ORBinf), left dorsolateral superior frontal gyrus (SFGdor), and some cerebellar regions, including the right cerebelum_crus1 and vermis_1_2 (Figure 2D and Supplementary Table 1).

In the right LBA, no significant differences were found in the RSFC between the MDD patients and HCs.

Additionally, hypo-connectivity was also found between the right SFA and some clusters of the bilateral putamen (Figure 2E and Supplementary Table 1).

### Correlations between baseline amygdala subregion RSFC and improvements in depressive symptoms in MDD patients

**Figure 3.**
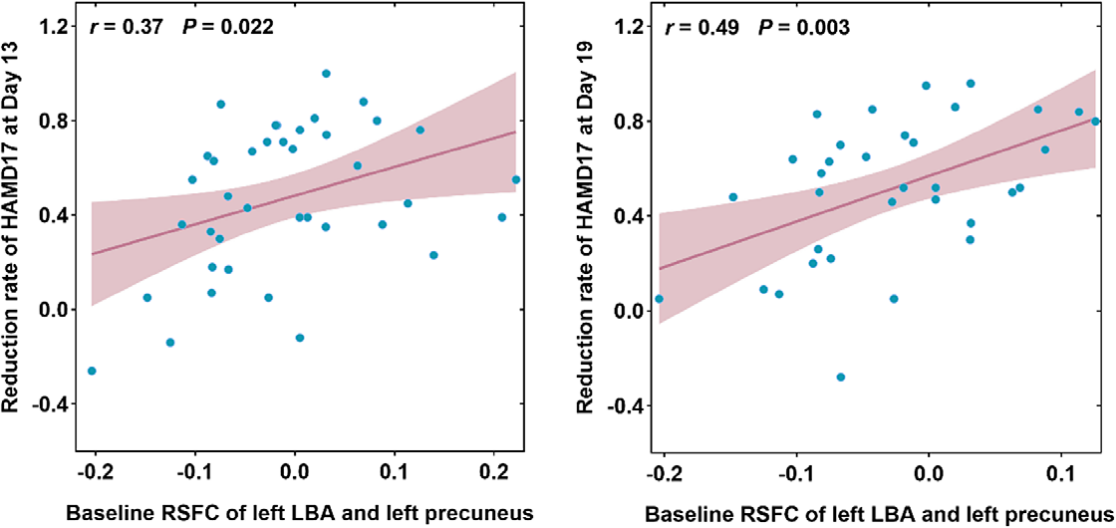
Correlations between improvements of depressive symptoms on Day 13 (A) and 19 (B) after ketamine treatment and abnormal amygdala subregion RSFC at baseline. HAMD-17, 17-item Hamilton Depression Scale; LBA, laterobasal; *r*, correlation coefficient; RSFC, resting-state functional connectivity.

At baseline, as shown in Figure 3A,B, the reduction rate of the HAMD-17 score on Day 13 (*r* = 0.37, *p* = 0.022) and the reduction rate of the HAMD-17 score on Day 19 (*r* = 0.49, *p* = 0.003) were positively correlated with the RSFC in the left LBA and left precuneus; even after increasing the medication load index as a covariate, this difference was still significant on Day 13 (*r* = 0.54, *p* = 0.001) and Day19 (*r* = 0.51, *p* = 0.002). No significant difference was found in the RSFC of other amygdala subregions on Day 13 or Day 19, regardless of whether the medication load index was added as a covariate.

### Predictive ability of amygdala subregion RSFC to predict the antidepressant effect of ketamine

After ketamine infusions, three regions, the left orbital part superior frontal gyrus (ORBsup), the right middle frontal gyrus (MFG), and the left ORBsup, exhibited a significant group × time interaction effect in the RSFC analysis (voxel-level *p* < 0.001, cluster-level *p* < 0.05 corrected by FDR), which used the left CMA, left LBA, and right CMA as seeds, respectively. In the analysis using the left LBA as a seed, we discovered a significant time main effect between responders and nonresponders in the region of the right MFG, left SFGdor, and right SOG (voxel-level *p* < 0.001, cluster-level *p* < 0.05 corrected by FDR); we also discovered a significant time main effect between responders and nonresponders in the region of the right MFG (voxel-level *p* < 0.001, cluster-level *p* < 0.05 corrected by FDR), which used the right SFA as a seed. All of the above results are shown in Table 2 and Supplementary Figure 1A–C.

**Table 2.** Regions showing significant differences in analysis

ROI	AAL	Effect	Clusters	MNI coordinates (peak)^a^			*f*-value	pFDR-corr
				*X*	*Y*	*Z*		
Left CMA	Left ORBsup	Interaction	60	−12	48	−21	24.19	0.001
Left LBA	Right MFG	Interaction	32	51	18	42	25.68	0.033
	Right MFG	Time	30	30	33	54	26.89	0.015
	Left SFGdor	Time	42	−15	60	18	23.96	0.005
	Right SOG	Time	41	36	−81	42	17.37	0.005
Right CMA	Left ORBsup	Interaction	40	−21	48	−21	27.50	0.012
Right SFA	Right MFG	Time	31	45	51	6	22.48	0.038

Abbreviations: AAL, anatomical automatic labeling; CMA, the centromedial amygdala; FDR, false discovery rate; LBA, the laterobasal amygdala; MFG, the middle frontal gyrus; ORBsup, the orbital part superior frontal gyrus; ROI, region of interest; SFA, the superficial amygdala; SFGdor, the dorsolateral superior frontal gyrus; SOG, the superior occipital gyrus.

a *X*, *Y*, *Z* – MNI (Montreal Neurological Institute) coordinates of significant effects.

**Figure 4.**
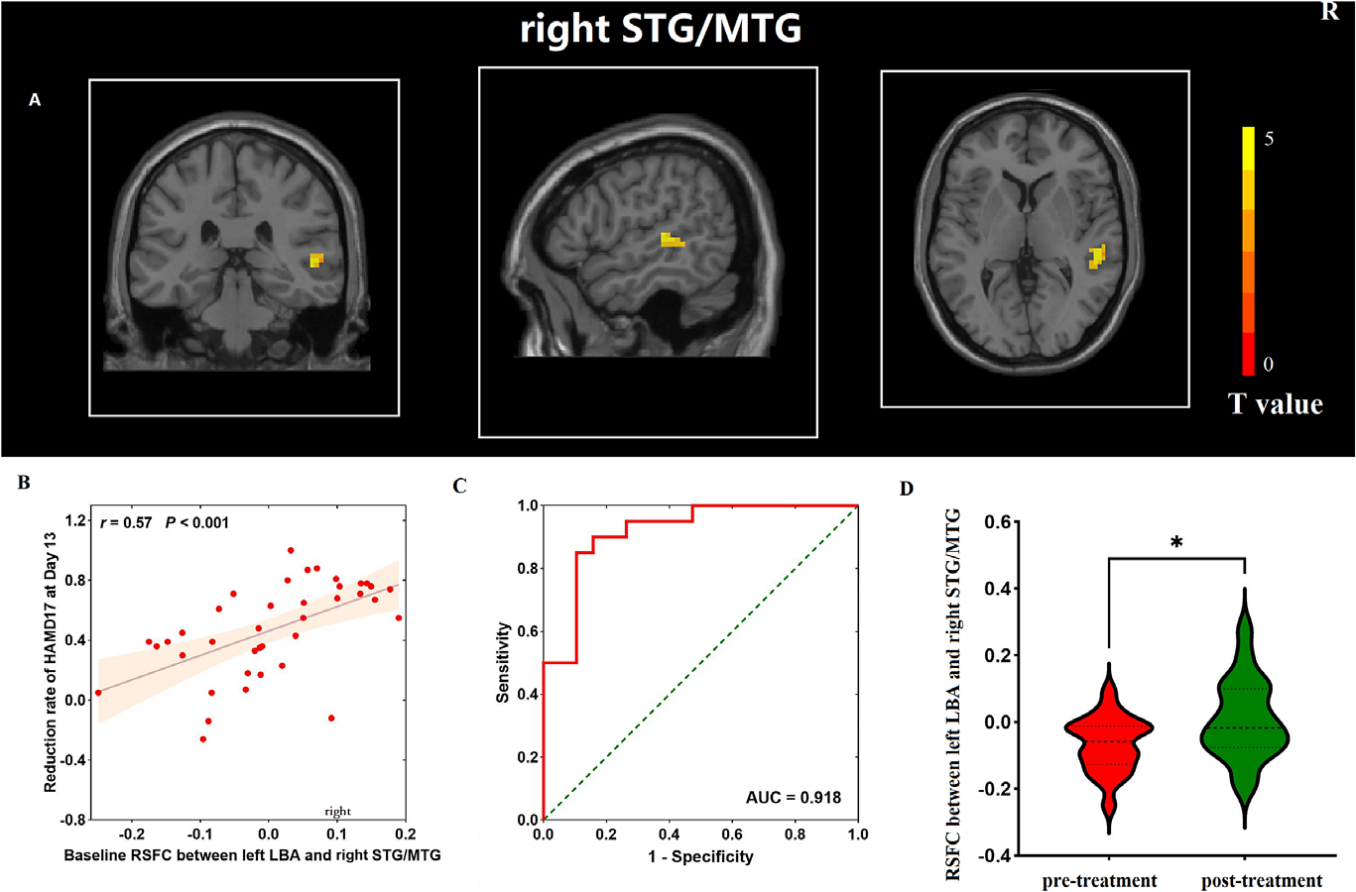
(A) Differences in the RSFC of the amygdala subregion between responders and nonresponders at baseline. (B) Correlation between the difference in amygdala subregion RSFC at baseline between responders and nonresponders and the reduction in depressive symptoms after ketamine treatment. (C) ROC curve analysis. The ROC curve showed an area under the curve (AUC) of 0.918 (95% confidence interval [CI] = 0.382–1.000, *p* < 0.001) for the RSFC of the left LBA and the right STG/MTG, with a sensitivity of 89.5% and specificity of 85.0%. (D) The difference in the RSFC of the left LBA and the right STG/MTG between nonresponders before and after treatment. AUC, area under the curve; HAMD-17, 17-item Hamilton Depression Scale; LBA, laterobasal; ROC, receiver operating characteristics; RSFC, resting-state functional connectivity; STG/MTG, superior temporal gyrus/middle temporal gyrus; *r*, correlation coefficient. *p-value < 0.05.

In addition, we assessed and compared whole-brain RSFC between responders and nonresponders. Compared with that of nonresponders, responders exhibited hyper-connectivity between the left LBA and the right superior temporal gyrus/middle temporal gyrus (STG/MTG) (cluster size = 33; Montreal Neurological Institute [MNI] coordinates: *x* = 51, *y* = −30, *z* = 3; *t*-value = 5.15, pFDR – corr <0.019; Figure 4A).

Bivariate correlation analysis revealed that the reduction rate of the HAMD-17 score on Day 13 was positively correlated with the baseline RSFC between the left LBA and right STG/MTG after ketamine treatment (*r* = 0. 57, *p* < 0. 001; Figure 4B). The ROC analysis indicated that the RSFC between the left LBA and right STG/MTG could predict the treatment response to ketamine on Day 13. The significant RSFC showed an effective differential capability with an area under the ROC curve of 0.918 (95% confidence interval = 0.832–1.000, *p* < 0.001; Figure 4C) for discriminating responders from nonresponders. The post hoc analysis conducted on nonresponder data revealed that the RSFC between the left LBA and right STG/MTG was significantly greater on Day 13 than at baseline after six ketamine infusions (−0.07 ± 0.08 vs. 0.01 ± 0.12, *t* = −2.660, *p* = 0.015; Figure 4D). No such findings were found among the responders.

## Discussion

Our study mainly explored the response-related changes in the RSFC of amygdala subregions induced by repeated ketamine infusions. In the present study, we revealed the following: (1) MDD patients displayed hyper-connectivity between the left LBA and the left precuneus, and the strength of this RSFC was positively correlated with the improvement of depressive symptoms after ketamine on Day 13 and Day 19; (2) the ketamine-induced response was related to changes in the RSFC between the bilateral CMA and the ORBsup and RSFC between the left LBA and the right MFG; and (3) the baseline RSFC between the left LBA and the right STG/MTG had the potential to predict the effect of ketamine in MDD patients.

Studies of MDD have shown that the precuneus is a key node of posterior default (pDMN), which plays a key role in the antidepressant treatment response. In this study, we found that the RSFC between the left LBA and the precuneus was stronger in MDD patients than in HCs, which was similar to the findings of previous studies [7, 30]. These studies revealed that the RSFC between the amygdala and the precuneus was stronger in grief or MDD participants. In addition, our study revealed that the stronger RSFC between the left LBA and the precuneus was related to the antidepressant effect of ketamine. This finding was similar to the result reported by Yuan et al. [22]. Their study showed that in all anxiety depression patients, the RSFC between the LBA and left precuneus of ketamine responders at baseline was stronger than that in nonresponders, and this RSFC was related to the reduction in depressive symptoms. In addition, in post hoc analysis, Yuan et al. reported that after ketamine infusions, the RSFC between the LBA and left precuneus was significantly reduced in responders. This finding was inconsistent with our findings. Our study revealed that the differences in efficacy between ketamine responders and nonresponders were mainly related to the ability of ketamine to regulate RSFC between amygdala subregions and prefrontal cortex (PFC). The use of different sample inclusion criteria and differences in application methodology may be important reasons for inconsistent conclusions. Nevertheless, another study has also emphasized the role of the precuneus circuit of the amygdala in treatment [31]. Considering the central role of the precuneus in self-referential thought and the positive correlation between the activation of the amygdala and rumination [32, 33], we speculated that the lower RSFC between the amygdala subregion and the precuneus may be the basis for MDD patients suppressing spontaneous negative self-concept at rest. According to our results, we believe that the stronger RSFC of the amygdala subregion and precuneus may be a marker of the antidepressant response to ketamine rather than a result of ketamine infusions. However, further studies with larger sample sizes are needed for verification.

Additionally, our study revealed that ketamine could extensively modulate the RSFC between amygdala subregions and the PFC. Numerous studies have confirmed the critical role of the prefrontal lobe in the treatment of MDD, and neuroanatomy has suggested that widespread connectivity from the PFC exerts top-down inhibitory control over the amygdala to regulate emotional expression [14, 34, 35]. Prefrontal cortex dysfunction is observed in treatment-resistant depression (TRD) patients and may contribute to the inability to control limbic areas such as the amygdala. Although current imaging studies have shown that ketamine treatment for MDD may be associated with modulation of the amygdala, to date, there has been no research exploring how ketamine affects amygdala functional pathways. Glutamate is an important neuroexcitatory transmitter in the amygdala and PFC, and another study reported that increased glutamatergic neurotransmission in the PFC is critical for the rapid antidepressant effects of ketamine [36]. The current study showed that a scaffold protein related to the glutamatergic pathway was altered in the PFC of MDD patients and that pathway activity was reduced [37]. At the same time, studies have confirmed decreased glutamate and its metabolites in regions such as the PFC of MDD patients [38, 39]. Milak et al. reported that the concentrations of glutamate and glutamine complexes in the PFC of the brains of patients who responded to ketamine treatment began to rise rapidly when ketamine was injected [40]. Thus we could reasonably speculate that ketamine may regulate brain communication between amygdala subregions and the PFC via the glutamate pathway.

Another important finding of this study was that the RSFC between the left LBA and right STG/MTG predicted the antidepressant efficacy of ketamine. The temporal lobe is involved in reward and affective processing [41, 42]. Structural imaging studies showed that the thickness of the isocortex of the temporal lobe increased significantly in TRD patients and that of the STG increased after modified electroconvulsive therapy (MECT) treatment [43]. A previous study reported decreased RSFC of the amygdala and temporal lobe in MDD patients and the decrease in RSFC between the amygdala and STG was related to increased rumination [44, 45]. One study also indicated that trauma could lead to a decrease in RSFC between the LBA and STG [46]. These studies suggested that there were structural and functional imbalances in the pathway of the amygdala and temporal lobe cortex in MDD patients. After subdividing the amygdala further, we also found RSFC abnormalities between the left LBA/SFA and temporal lobe. Notably, we found that responders had stronger RSFC between the left LBA and right STG/MTG, whereas in post hoc analyses, the RSFC between the left LBA and right STG/MTG of nonresponders was significantly stronger after ketamine treatment. A previous repetitive transcranial magnetic stimulation (rTMS) study revealed that glucose metabolism in the left fusiform and left MTG after treatment was significantly reduced only in rTMS responders, and nonresponders showed a worsening pattern of increased metabolism in the bilateral temporal cortex and fusiform gyrus [47]. These findings may indicate that ketamine may have a therapeutic effect through a mechanism that modulates hyperactivity normalization in the STG/MTG.

Finally, several limitations need to be considered. First, the sample size was small, which was one of the most important shortcomings of our study. Second, participants received ketamine infusions with concurrent antidepressant medications, which might affect the observed response to ketamine, but an add-on ketamine study was able to provide real-world data. Moreover, we calculated a composite measure of the total medication load for each individual (reflecting the dose and variety of different medications taken) by summing all individual medications and adding this index as a covariate during the statistical analysis [48, 49], which further enhanced the persuasiveness of the research conclusions. We are currently conducting a randomized controlled study with a larger sample size, and we hope that subsequent analysis can verify the conclusions of the study or provide additional research findings.

This study suggested that the mechanism by which ketamine improved depressive symptoms may be related to its regulation of RSFC in the amygdala subregion; at the same time, our study supported that the RSFC of the amygdala subregion may be a predictor of antidepressant neurologic responses to ketamine treatment. Future studies should further confirm the potential of these neural targets to predict therapeutic efficacy with a larger sample of randomized, placebo-controlled studies.

## Supporting information

Liu et al. supplementary material Liu et al. supplementary material

## Data Availability

The data included in this study are available from the corresponding author upon reasonable request.

## Supplementary material

The supplementary material for this article can be found at http://doi.org/10.1192/j.eurpsy.2024.1744.
