# Supplementary material for: Functional connectivity of the amygdala subregions and the antidepressant effects of repeated ketamine infusions in major depressive disorder
Source: Eur Psychiatry. 2024 Apr 4;67(1):e33. doi: 10.1192/j.eurpsy.2024.1744 (PMC11059247; doi:10.1192/j.eurpsy.2024.1744)
Supplement: Liu et al. supplementary material [file S0924933824017449sup001.docx]

Supplementary material

Supplementary Table: 1

Supplementary Figure:1

**Supplementary table 1.** Differences in the RSFC of amygdala subregions between HCs and MDD patients.

| **ROI** | **clusters** | **AAL** | **T value** | **MNI coordinates (Peak)^a^** | | | **pFDR-corr** |
| --- | --- | --- | --- | --- | --- | --- | --- |
|  |  |  |  | **X** | **Y** | **Z** |  |
| **left CMA** |  | **HC > MDD** | | | | | |
|  | 113 | left insula | 5.33 | -33 | 15 | -3 | p<0.001 |
|  | 175 | right insula | 5.29 | 30 | -18 | -3 | p<0.001 |
|  | 126 | left putamen | 6.25 | -30 | -18 | -6 | p<0.001 |
|  | 35 | right SMA | 4.55 | 9 | 12 | 45 | 0.043 |
|  |  | **MDD > HC** | | | | | |
|  | 120 | left postcentral gyrus | 4.67 | -42 | -36 | 66 | p<0.001 |
| **left LBA** |  | **MDD > HC** | | | | | |
|  | 87 | left precuneus | 4.41 | -9 | -75 | 54 | 0.001 |
|  | 56 | right inferior temporal gyrus | 4.73 | 57 | -39 | -24 | 0.005 |
| **left SFA** |  | **HC > MDD** | | | | | |
|  | 191 | right putamen | 5.55 | 33 | 6 | 3 | p<0.001 |
|  | 105 | right DCG | 4.57 | 9 | 15 | 42 | p<0.001 |
|  | 53 | left putamen | 3.84 | -24 | 18 | 6 | 0.010 |
|  | 38 | left IFGoperc | 4.43 | -51 | 9 | 6 | 0.033 |
| **right CMA** |  | **HC > MDD** | | | | | |
|  | 330 | right cerebelum_crus1 | 5.45 | 39 | -57 | -36 | p<0.001 |
|  | 123 | vermis_1_2 | 4.89 | 3 | -45 | -24 | p<0.001 |
|  | 53 | left ORBinf | 5.57 | -33 | 33 | -9 | 0.011 |
|  | 46 | left SFGdor | 4.58 | -24 | 60 | 21 | 0.016 |
|  |  | **MDD > HC** | | | | |  |
|  | 34 | right rolandic operculum | 4.47 | 42 | -24 | 21 | 0.048 |
|  | 72 | right superior occipital gyrus | 5.03 | 24 | -84 | 36 | 0.002 |
|  | 173 | left postcentral gyrus | 4.66 | -54 | -18 | 51 | p<0.001 |
|  | 90 | right postcentral gyrus | 4.22 | 48 | -30 | 60 | 0.001 |
|  | 110 | left PCL | 5.08 | 0 | -30 | 60 | p<0.001 |
|  | 111 | right postcentral gyrus | 5.31 | 30 | -39 | 69 | p<0.001 |
| **right SFA** |  | **HC > MDD** |  |  |  |  |  |
|  | 62 | right putamen | 5.00 | 33 | 6 | 3 | 0.005 |
|  | 100 | left putamen | 6.06 | -18 | -3 | 15 | 0.001 |

Abbreviations: RSFC: resting-state functional connectivity; MDD: Major depressive disorder; HCs: healthy controls; ROI: region of interest; AAL: Anatomical Automatic Labeling; FDR: false discovery rate.

SMA: the supplementary motor area; DCG: the median cingulate and paracingulate gyri; IFGoperc: the opercular part inferior frontal gyrus; ORBinf: the orbital part inferior frontal gyrus; SFGdor: the dorsolateral superior frontal gyrus; PCL: the paracentral lobule.

a: x, y, z = MNI (Montreal Neurological Institute) coordinates of significant effects.


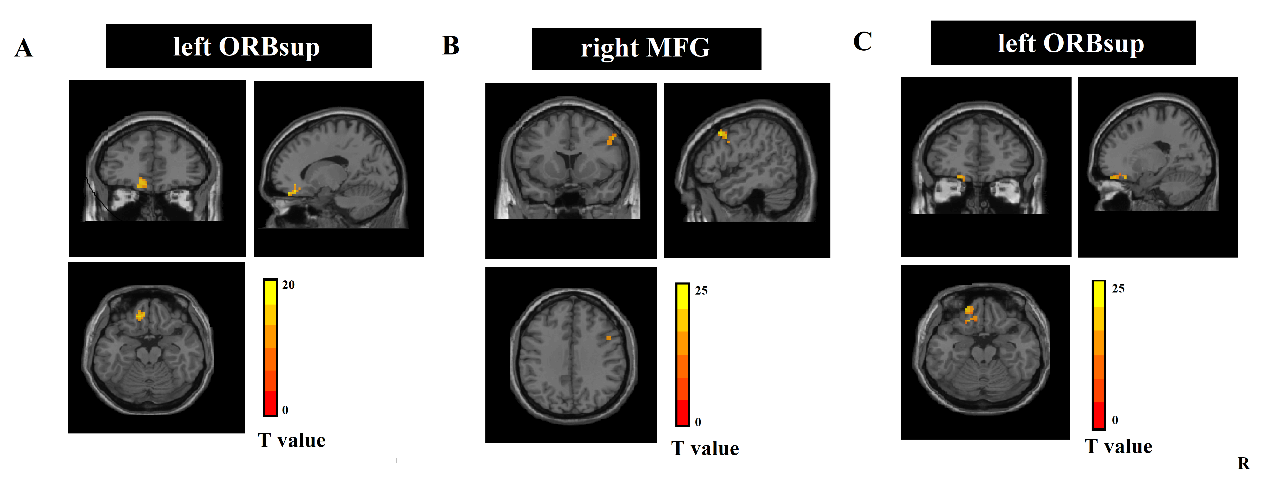


**Supplementary Figure 1A-C** Regions showing significant group-by-time interaction effect in analysis (voxel-level p < 0.001, cluster-level p < 0.05 corrected by FDR). ORBsup：the orbital part superior frontal gyrus; MFG: the middle frontal gyrus. R: right.
